# Biomechanical Comparison of Hybrid Technique and Traditional Dual-Growing Rods Alone in the Treatment of Severe Early Onset Scoliosis

**DOI:** 10.3390/jcm15145352

**Published:** 2026-07-08

**Authors:** Chenkai Li, You Du, Hanming Bian, Yang Yang, Guanfeng Lin, Yiwei Zhao, Xiaohan Ye, Jianguo Zhang, Shengru Wang

**Affiliations:** Department of Orthopedic Surgery, Peking Union Medical College Hospital, Peking Union Medical College and Chinese Academy of Medical Sciences, 1st Shuai Fu Yuan, Dongcheng District, Beijing 100730, Chinabhmspine@163.com (H.B.);

**Keywords:** early-onset scoliosis, traditional dual-growing rods, hybrid technique, finite element analysis, in vitro biomechanical experiments

## Abstract

**Background:** Currently, there is a lack of biomechanical studies on traditional dual-growing rods (TDGR) combined with apical osteotomy and short-segment fusion (hybrid technique, HT). This study compared the differences in clinical outcomes and biomechanics between TDGR and HT in the treatment of severe early-onset scoliosis (sEOS) via finite element analysis (FEA) and in vitro biomechanical experiments. **Methods:** Two scoliotic FEA models and 12 in vitro scoliotic models were created. In the FEA, the initial surgeries for TDGR and HT, two subsequent lengthenings, and up to 12 months of physeal spinal growth were simulated. In the in vitro biomechanical experiments, the initial surgeries were simulated. Correction outcomes, spinal height, and stress were compared between the TDGR and HT groups. **Results:** (1) FEA: Compared with TDGR, HT achieved better correction (62.4% vs. 36.2%) and a greater increase in spinal height (26.43 mm vs. 12.58 mm) after the initial surgery. During follow-up, HT resulted in better correction maintenance and could better sustain spinal growth than TDGR. HT reduced the stress on the proximal and distal instrumented vertebral bodies, junctional intervertebral discs, and instrumentation compared with TDGR. (2) In vitro biomechanical experiment: After the initial surgery, the mean Cobb angle of the main curve (24.58 ± 2.80° vs. 38.97 ± 3.23°) and AVT (8.87 ± 1.64 mm vs. 13.15 ± 3.58 mm) in the HT group were significantly lower than those in the TDGR group (*p* < 0.05). The increase in spinal height in the HT group was significantly greater than that in the TDGR group (3.83 ± 0.45 cm vs. 1.85 ± 0.72 cm, *p* < 0.001). Compared with TDGR, HT significantly decreased rod strain (*p* < 0.05). **Conclusions:** Compared with TDGR, HT can significantly improve correction outcomes and maintain spinal growth. Apical anchors can effectively disperse stress on the spine and instrumentation, which may reduce the risk of complications and potentially delay intervertebral disc degeneration, although clinical validation is required.

## 1. Introduction

Severe early-onset scoliosis (sEOS) refers to spinal deformities caused by various factors before the age of 10, with a main curve Cobb angle > 80° [1,2]. Given that sEOS patients are immature with short spinal heights and limited thoracic volumes, the inhibition of growth and development at this stage can cause irreversible damage to cardiopulmonary function. Therefore, the treatment principle for sEOS patients is to correct the deformity while maintaining spinal and thoracic development [3]. On the basis of this principle, growth-friendly techniques have become mainstream treatment options, with the traditional growing rods (TGRs) being the most widely used treatment in clinical practice [4,5].

In traditional dual-growing rod (TDGR) constructs, the proximal and distal anchors bear the majority of the distraction forces, while the apical region remains unsupported. This configuration creates a long lever arm, resulting in high stress concentrations at the foundation implants and the rod–connector junctions. Clinical series have reported mechanical complication rates ranging from 29% to 72% in TDGR treatment, with proximal junctional kyphosis (PJK) and implant failure being the most frequently observed adverse events [6,7]. Consequently, TDGR treatment often leads to insufficient correction of the main curve, and postoperative progression of apical vertebral translation and rotation is frequently observed, further increasing the risk of mechanical complications. To address these limitations, Wang et al. [8] proposed combining TDGR with apical osteotomy and short-segment fusion (hybrid technique, HT). Previous clinical studies have reported that HT not only significantly enhances correction outcomes but also decreases the incidence of mechanical complications by dispersing stress on the spine and instrumentation [9,10]. However, all current studies on HT are clinical, with limited sample sizes and high heterogeneity. Moreover, directly measuring in vivo stresses on the spine and implants is impossible and unethical, leaving the actual contribution of apical osteotomy and short-segment fusion to stress dispersion unknown. While independent studies have explored other apical control strategies, such as the Shilla technique and apical pedicle screws [11,12], the biomechanical mechanism of combining apical osteotomy with dual-growing rods has not been directly investigated. Although finite element analysis (FEA) has been employed to simulate distraction forces and identify potential implant failure zones in traditional growing rod constructs [13,14], no FEA study has directly compared the biomechanical profile of HT with that of TDGR under identical simulated growth conditions.

We therefore hypothesised that HT would: (1) achieve superior initial correction and better maintain correction during follow-up by eliminating the asymmetric growth potential of the apex, and (2) effectively redistribute stress from the proximal and distal anchors and rod–connector junctions to the apical anchors, thereby reducing peak stresses on the instrumentation and adjacent intervertebral discs. Therefore, to test these hypotheses, this study compared the effects of HT and TDGR on correction outcomes, spinal height, and biomechanics via finite element analysis (FEA) and in vitro biomechanical experiments in highly homogeneous sEOS models.

## 2. Materials and Methods

### 2.1. Scoliosis Model

#### 2.1.1. Finite Element Analysis

Based on the CT data of a 9.6-year-old girl (congenital scoliosis, T10 wedge vertebra, weight 45.8 kg, height 138 cm, coronal Cobb angle 84.3°, and sagittal Cobb angle 42.9°), a patient-specific, ligamentous, scoliotic finite element model of T1–L5 was established. The model was validated using three approaches (detailed in Figures S1 and S2):

(1) Geometric validation: The main curve Cobb angle in the finite element model was 82.4°, compared to 84.3° on the patient’s standing radiograph (difference 1.9°).
(2) Supine bending validation: The displacement of each vertebral centroid from the L5 midline in the model under simulated left/right bending showed good agreement with the bending radiographs (mean absolute difference <2.1 mm).
(3) Segmental range of motion and stiffness: The flexion–extension, lateral bending, and axial rotation ranges for the T1–T4 and L1–L4 segments, as well as the average stiffness of T12–L2 under vertical loading, flexion, extension, lateral bending, and rotation, were compared with published cadaveric data [15,16,17,18]. All simulated values fell within the range of previously reported experimental results (Table S1).

These validation steps confirm that the model accurately represents the preoperative scoliotic geometry and spinal kinematics.

#### 2.1.2. In Vitro Biomechanical Experiment

Twelve fresh-frozen ovine spines (T1–L4) from one-year-old sheep were obtained from a licenced commercial supplier for in vitro biomechanical experiments. All the specimens were subjected to radiological assessment to exclude specimens with vertebral fractures, congenital deformities, or other structural abnormalities. The specimens were wrapped in double-layer plastic film and stored at −20 °C. Before modelling and testing, the specimens were thawed at room temperature (22–25 °C) for 3 h. The in vitro scoliotic model was created according to the method reported by Wilke et al. [19]. Both modelling and testing were conducted at room temperature, with specimen humidity maintained throughout the process.

### 2.2. Simulation of Surgery and Physeal Spinal Growth

#### 2.2.1. Finite Element Analysis

The models of pedicle screws and fixation rods were constructed using SolidWorks 2020 software (v2020). After assembly, they were imported into Ansys 2023 R1 software (v2023r1) for assigning relevant material properties. The materials and material parameters are shown in Table S1.

TDGR and HT (TDGR + T10/11 intervertebral osteotomy and T9–T11 internal fixation) were simulated. TDGR implantation was as follows: the pedicle screws were inserted proximally (T3–4) and distally (L3–4). Two long rods were placed on the proximal anchors, and two short rods were placed on the distal anchors. The proximal and distal rods were then connected with side-to-side connectors. Then, the distraction was simulated, and the rods were fixed within the connectors. Both models had identical distraction displacements of 20 mm. The TDGR model comprised approximately 286,187 nodes and 1,196,049 elements.

In the HT group, the pedicle screws were initially inserted (T9–T11). Then, the T10 inferior spinous process, T11 and T12 superior spinous process, T10 inferior 1/2 lamina and bilateral inferior facet, bilateral T11 superior facet, and T10/11 intervertebral disc and upper and lower bony endplates were removed. The osteotomy gap was subsequently closed via compression. TDGR implantation was then performed as described. The HT model comprised approximately 354,342 nodes and 1,456,010 elements.

During each distraction, the connectors were uncoupled, and then the fixation rods were distracted by 10 mm towards the cranial and caudal directions. Finally, the connector was locked.

Based on previous studies, spinal growth was simulated via the following formula: *G = G_m_ [1 − β(σ − σ_m_)]* [13,20,21]. Here, *G* represents the actual vertebral growth, *G_m_* represents the baseline growth rate (*G_m_* = 1 mm/year), *σ* represents the actual stress on the vertebra, *σ_m_* represents the baseline stress (*σ_m_* = 0.5 MPa), and *β* represents a constant *β* = 1.3 MPa^−1^. This linear mechanobiological model assumes that stress inversely modulates growth, a simplification commonly used in comparative FEA studies of growing rods [13,20,21]; it is best suited for directional comparisons rather than absolute growth prediction.

The material properties of the current models were determined according to previous studies (Table S1) [14]. All osseous and soft tissues were modelled as linear elastic isotropic materials, which is acceptable for the relatively small deformations occurring under distraction loads, as supported by previous FEA studies of growing rod constructs [14]. Ligaments were modelled as spring elements with published stiffness coefficients (Table S1). The annulus fibrosus was modelled as a ground substance without fibre reinforcement, a common simplification in comparative spine FEA. These assumptions are consistent with prior validated models [22] and do not compromise the directional comparison between TDGR and HT. In each model, the effects of gravity and paraspinal muscles were simulated. The gravitational acceleration was set at 9.8 m/s^2^, and the loads on each vertebra were determined based on a previous study (Table S2) [23].

#### 2.2.2. In Vitro Biomechanical Experiment

Initial TDGR and HT surgeries were simulated referring to the methods described in the FEA.

### 2.3. Parameter Analysis

#### 2.3.1. Finite Element Analysis

The outcomes were evaluated via radiological and biomechanical parameters. The radiological parameters included the Cobb angle of the main curve, apical vertebral translation (AVT), global kyphosis, and T1–L5 height. The biomechanical parameters included junctional intervertebral disc stress (T2/3 and L4/5), instrumented vertebral body stress at the apex (T9, T10, and T11) as well as at the proximal and distal anchors (T3, T4, L3, and L4), screw stress at the proximal and distal anchors (T3, T4, L3, and L4), and rod stress. In both models, initial surgery, 2 subsequent lengthenings, and up to 12 months of physeal spinal growth were simulated.

#### 2.3.2. In Vitro Biomechanical Experiment

The outcomes were evaluated via radiological and biomechanical parameters. The radiological parameters included the Cobb angle of the main curve, AVT, and T1–L5 spinal height. Biomechanical parameters referred mainly to the strain of the rods. Strain gauges were placed at the midpoints of the four rods. After the initial correction, the strain of each rod was recorded. The model was subsequently fixed to the testing machine (Shenzhen SUNS Technology Stock Co., Ltd., Shenzhen, China). An axial load of 100 N was applied to simulate the load on the vertebrae, and the strain of each rod was recorded (Figure S3). This load level approximates the upper body weight supported by the spine in a small child (approximately 10 kg) during standing, is within the range used in previous in vitro paediatric spine biomechanical studies and produces measurable strain without damaging the specimens. Each model was tested three times, with the results of the third test being taken.

### 2.4. Statistical Analysis

Analysis was performed via SPSS 27.0 (IBM). Continuous variables are presented as means ± standard deviations. Student’s *t*-test was used to compare continuous variables. A *p* < 0.05 was considered statistically significant. For the finite element analysis (FEA), because the model is deterministic (identical geometry, boundary conditions, and material properties for both TDGR and HT simulations), stress and deformation outputs are single-valued at each time point without inter-sample variability. Therefore, inferential statistics were not applied to the FEA results. For the in vitro biomechanical experiments (*n* = 6 per group), effect sizes (Cohen‘s d) and 95% confidence intervals for the mean differences are reported in Table S3.

## 3. Results

### 3.1. Scoliotic Model

#### 3.1.1. Finite Element Analysis

The final FEA models included 286,187 (TDGR) and 354,342 (HT) nodes and 1,196,049 (TDGR) and 1,456,010 (HT) elements. A formal mesh convergence study was not performed independently for this study. However, the mesh density used (approximately 1.2 million elements for TDGR and 1.45 million for HT) is consistent with, or higher than, that in previous finite element studies of scoliosis with growing rod constructs [13,14]. Because the same mesh topology, element types, and boundary conditions were applied to both TDGR and HT simulations, any systematic discretization error would affect both models equally, thus preserving the validity of comparative conclusions.

#### 3.1.2. In Vitro Biomechanical Experiment

The mean Cobb angle of the main curve and the mean AVT were 81.33 ± 3.85° and 64.43 ± 13.40 mm, respectively. The mean overall spinal height was 49.97 ± 1.80 cm (Figure S4).

### 3.2. Parameter Analysis

#### 3.2.1. Finite Element Analysis

Compared with the TDGR group, the HT group presented better coronal and sagittal correction. After the initial surgery, the increase in T1–L5 height was greater in the HT group than in the TDGR group. However, during follow-up, the increase in T1–L5 height was greater in the TDGR group than in the HT group. By the last follow-up, the T1–L5 height in the HT group was still greater than that in the TDGR group (Table 1).

**Figure 1:**
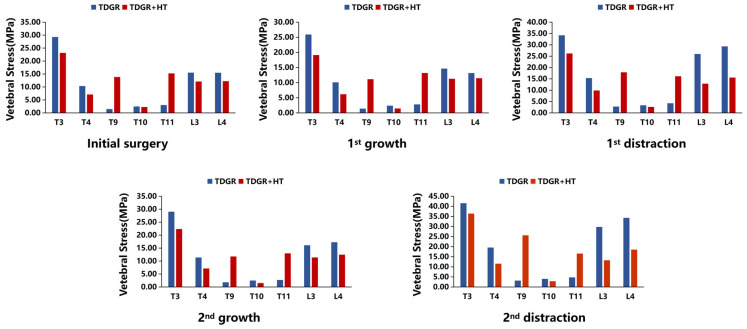
Comparison of the stress on the apical vertebrae between the TDGR and HT groups.

**Figure 2:**
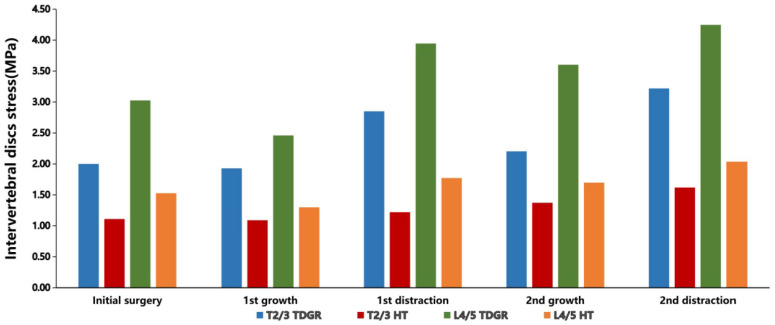
Comparison of the stress on the junctional intervertebral discs between the TDGR and HT groups.

**Figure 3:**
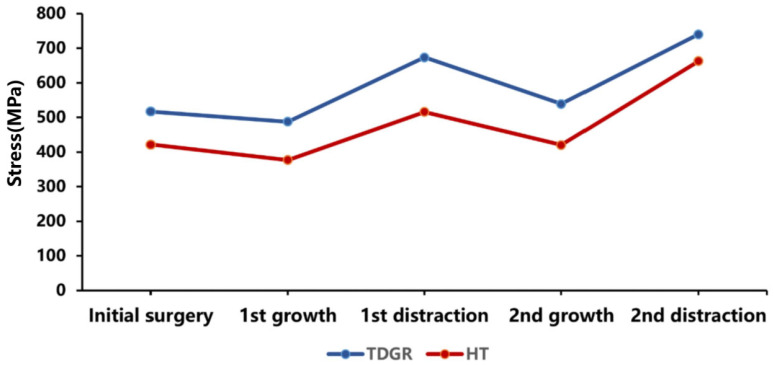
Comparison of the maximum stresses on the proximal and distal anchors between the TDGR and HT groups.

**Figure 4:**
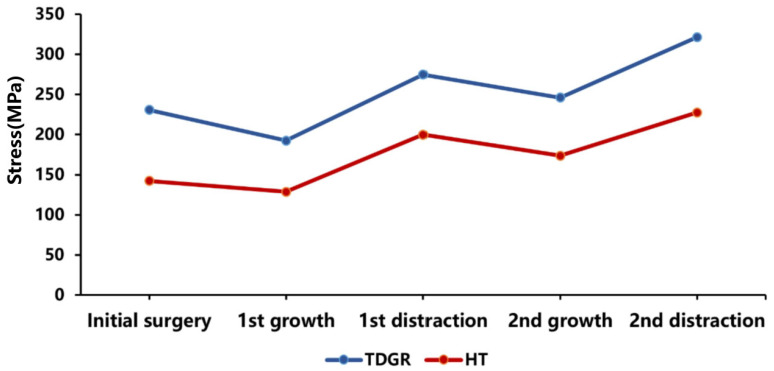
Comparison of the maximum stress on the rods between the TDGR and HT groups.

The stress on the proximal and distal instrumented vertebrae was greater in the TDGR group than in the HT group; the stress of the apical vertebrae was greater in the HT group than in the TDGR group (Figure 1 and Table S4). Additionally, compared with TDGR, HT reduced stress on the junctional intervertebral discs (Figure 2 and Table S5). In the TDGR group, the maximum stresses on the proximal and distal anchors at each follow-up were 516.69 MPa, 487.36 MPa, 673.10 MPa, 538.91 MPa, and 739.91 MPa, respectively. In the HT group, the corresponding maximum stress were 421.14 MPa, 376.74 MPa, 515.62 MPa, 420.18 MPa, and 662.39 MPa, respectively (Figure 3 and Figure S5 and Table S6). In the TDGR group, the maximum stress on the rods at each follow-up were 230.79 MPa, 192.50 MPa, 274.69 MPa, 246.01 MPa, and 321.45 MPa, respectively. In the HT group, the maximum stress on the rods were 142.27 MPa, 128.66 MPa, 199.95 MPa, 173.77 MPa, and 227.45 MPa, respectively (Figure 4 and Figure S6 and Table S7).

#### 3.2.2. In Vitro Biomechanical Experiment

**Figure 5:**
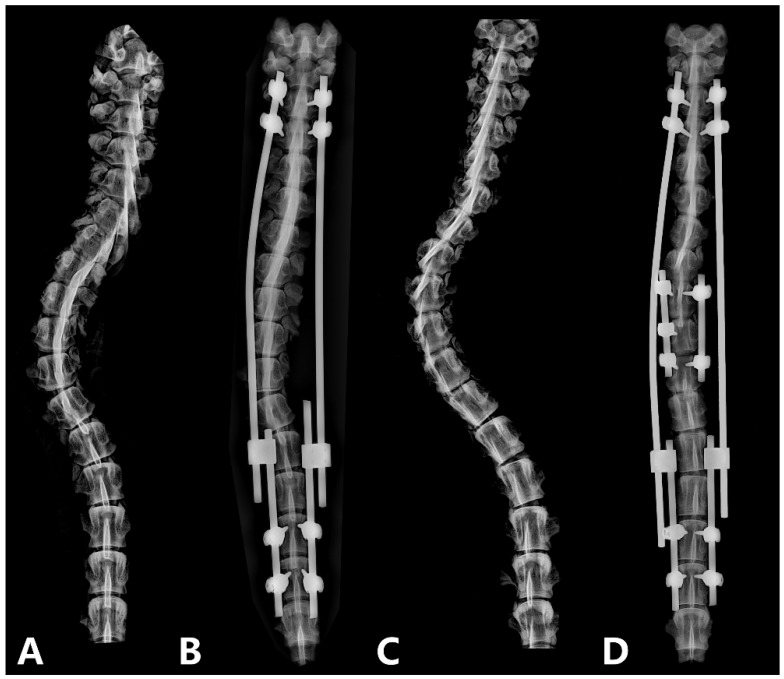
Preoperative and postoperative radiographs of TDGR and HT, (**A**) preoperative radiographs of TDGR; (**B**) postoperative radiographs of TDGR; (**C**) preoperative radiographs of HT; (**D**) postoperative radiographs of HT.

No significant differences were observed between the TDGR and HT groups in terms of the preoperative severity of the deformity or the T1-L5 height (*p* > 0.05). After the initial surgery, the mean Cobb angle of the main curve and AVT in the HT group were significantly lower than those in the TDGR group (HT vs. TDGR: main curve, 24.58 ± 2.80° vs. 38.97 ± 3.23°, *p* < 0.001; AVT, 8.87 ± 1.64 mm vs. 13.15 ± 3.58 mm, *p* = 0.035). Moreover, compared with the TDGR group, the HT group presented a greater increase in T1-L5 height (HT vs. TDGR: 3.83 ± 0.45 cm vs. 1.85 ± 0.72 cm, *p* < 0.001) (Table 2 and Figure 5).

After initial correction, the rod strain in the TDGR group was lower than that in the HT group, but the difference was not significant (*p* > 0.05). After the application of a 100 N axial load, the increase in rod strain in the TDGR group was significantly greater than that in the HT group (*p* < 0.05). Moreover, the strains of the right lower, left upper, and right upper rods in the HT group was significantly lower than that in the TDGR group (*p* < 0.05) (Table 3).

## 4. Discussion

This study compared the biomechanical performance of HT and TDGR in the treatment of sEOS using both FEA and in vitro experiments. The results demonstrated that HT achieved superior correction outcomes, better maintained spinal height, and effectively reduced stress on the instrumentation and adjacent structures. Below, we discuss these findings in the context of existing literature and their potential clinical implications.

### 4.1. Correction Outcomes and Spinal Height Maintenance

Previous studies have shown that compared with TDGR, HT can significantly improve correction outcomes and decrease the incidence of complications [24]. Based on the clinical results, we hypothesised that the potential mechanisms are as follows: On the one hand, the apical osteotomy and short-segment fusion not only enhance the correction outcomes after the initial surgery but also eliminate the significant asymmetric growth potential at the apex, thereby improving correction maintenance during follow-up. On the other hand, the apical anchors increase the number of stress distribution points, whereas short-segment fusion at the apex enhances both local and general spinal stability, thereby effectively reducing and dispersing the biomechanical stress on the spine and implants. However, there is currently no biomechanical analysis regarding TDGR and HT. FEA is an efficient numerical computation method that simulates real physical systems through mathematical approximation to solve practical problems in complex structures, including those in the field of mechanics [25,26,27]. Moreover, animal spinal specimens, such as sheep spines, are highly similar to human spines in terms of tissue structural properties and can be used to effectively simulate the biomechanical state of the human spine under specific conditions when subjected to different loads. Therefore, for the first time, the present study evaluated the differences in clinical outcomes and biomechanics between TDGR and HT by combining FEA with in vitro biomechanical experiments. Unlike previous clinical reports that were limited by small sample sizes, high heterogeneity, and an inability to measure in vivo stresses, this study provides direct biomechanical evidence using highly homogeneous models and controlled experimental conditions. The results revealed that: (1) compared with TDGR, HT achieved better correction and correction maintenance; (2) compared with TDGR, HT maintained spinal growth; and (3) apical anchors helped to disperse the stress on the proximal and distal instrumented vertebral bodies, the junctional intervertebral discs, and the instrumentation.

In terms of correction, the FEA results revealed that at the initial correction, the correction rate of the main curve in the HT group was greater than that in the TDGR group (HT vs. TDGR: 62.4% vs. 36.3%), and AVT correction was also greater (HT vs. TDGR: 19.2 mm vs. 10.4 mm). During subsequent follow-up, correction maintenance was better in the HT group than in the TDGR group. Similarly, the in vitro biomechanical experiments also indicated that after initial correction, the Cobb angle of the main curve and AVT in the HT group were significantly lower than those in the TDGR group (*p* < 0.05). The results revealed that compared with TDGR, HT not only improved the correction outcomes of initial surgery but also improved correction maintenance during follow-up. This finding is consistent with previous clinical research [9,10,24]. Apical osteotomy and short fusion significantly improved correction and prevented correction loss. On the one hand, compared with TDGR, HT allows surgeons to perform more correction manoeuvres at the apex in the initial surgery to achieve powerful mechanical correction. On the other hand, in addition to mechanical correction, apical osteotomy can eliminate the enormous asymmetric growth potential of the apex, which can prevent deformity progression similar to the crankshaft phenomenon during follow-up, thereby improving correction maintenance.

Whether apical anchors negatively affect spinal growth remains a concern. By evaluating patients who had graduated from TDGR and HT treatments, our previous study revealed that HT can maintain the growth of the spine [9,24]. In the present study, the FEA results revealed that the increase in T1-L5 height after initial correction was greater in the HT group than in the TDGR group (HT vs. TDGR: 26.43 mm vs. 12.58 mm). Similarly, in vitro biomechanical experiments indicated that at the initial surgery, the increase in T1–L5 height was significantly greater in the HT group than in the TDGR group (HT vs. TDGR: 3.83 ± 0.45 cm vs. 1.85 ± 0.72 cm, *p* < 0.001). However, the trend in the increase in T1-L5 height reversed during follow-up. By the last follow-up, the T1-L5 height in the HT group remained greater than that in the TDGR group. This finding indicated that although HT may have little impact on spinal growth, this impact is not significant and can be partially compensated for by greater increase in spinal height at the initial surgery. The results also demonstrate the importance of powerful correction at the initial surgery for maintenance of the final spinal height. The combination of clinical studies and current biomechanical research provides biomechanical evidence supporting that HT can effectively improve correction outcomes while maintaining spinal growth. Notably, the impact of HT on spinal growth needs to be assessed holistically, rather than being confined solely to the apical fusion segment. We believe that for sEOS patients, arresting the growth of the apex outweighs its disadvantages. On the one hand, the growth at the apex is asymmetric, and without intervention, the progression of deformity caused by the growth of the apex will lead to greater loss of spinal height rather than an increase in height. On the other hand, through apical osteotomy and short-segment fusion, HT achieves a greater increase in spinal height at the initial surgery than does natural growth of the corresponding segment.

### 4.2. Stress Distribution and Implant-Related Complications

Implant- and alignment-related complications are common mechanical complications following TDGR treatment. Studies have shown that these mechanical complications are related to the correction rate and stress concentration on the instrumentation [28]. This is because a larger residual curve can lead to greater stress on the spine and instrumentation. Furthermore, sEOS patients have substantial asymmetric growth potential in the apex. In the absence of apical control, postoperative deformity progression exacerbates stress concentration on the spine and instrumentation. Moreover, prolonged stress concentration can lead to metal fatigue, increasing the risk of implant fracture, as well as bone resorption at the anchors. Multiple factors interact with each other, thereby increasing the risk of implant loosening and displacement. Additionally, stress concentration at junctional zones can increase the risk of junctional kyphosis and other alignment-related complications [22]. The present results revealed that apical anchors can effectively improve correction outcomes and disperse the stress on instrumented vertebrae and instrumentation, thereby decreasing the risk of implant-related complications such as rod breakage and screw loosening. Moreover, by decreasing the stress on the junctional vertebrae and intervertebral discs, HT may also help reduce the risk of alignment-related complications. Notably, the ability of HT to disperse biomechanical stress on the spine and instrumentation may be underestimated in the current study. To control for confounding factors, the same distraction distance was applied during initial correction by both TDGR and HT in the FEA and in vitro biomechanical experiments. However, in the clinical treatment of sEOS, TDGR often requires greater distraction than HT to achieve satisfactory correction, which would further exacerbate the stress concentration on the spine and instrumentation.

One notable finding of this study is that apical vertebral stresses were higher in the HT group than in the TDGR group (e.g., maximum stress at T9: 25.63 MPa in HT vs. 3.17 MPa in TDGR; see Table S3). This increase is expected because the apical anchors now bear additional loads. Importantly, these stress levels are substantially lower than the reported [29] compressive strength of paediatric cortical bone, indicating that the risk of acute vertebral fracture is low. Furthermore, in our clinical HT series, no apical screw loosening or vertebral body fracture was observed, providing indirect support for the safety of this stress redistribution. Nevertheless, caution is advised in very young patients or those with compromised bone quality, and long-term follow-up of bone remodelling around apical anchors is warranted. The peak anchor stress in the TDGR group (739.91 MPa) is below the yield strength of Ti-6Al-4V (880–950 MPa) but approaches its fatigue limit. This level of stress, combined with cyclic loading, may increase the risk of rod fracture over time. In contrast, the HT group exhibited lower peak stresses (662.39 MPa), suggesting a reduced fatigue risk. Additionally, high implant stresses may contribute to screw loosening through overload at the bone–implant interface. These findings are consistent with the higher mechanical complication rate observed in TDGR-treated patients in our clinical series [24].

### 4.3. Impact on Junctional Intervertebral Discs

Previous studies have shown that intradiscal stress in the range of 1–3 MPa is conducive to stimulating the synthesis of proteoglycans. However, when the intradiscal stress is >3 MPa, it may have a negative impact on the physiological function of the intervertebral disc [30,31,32]. Additionally, Shiri et al. [33] reported that intervertebral disc degeneration was accelerated by increased loads. In the present study, the FEA results demonstrated that compared to TDGR, HT reduced the stress on the junctional intervertebral discs and maintain the load on the intervertebral discs within the physiological range, which may help maintain the health of the intervertebral discs in the noninstrumented segments and delay the degeneration of the intervertebral disc. Moreover, since the lumbar spine plays an important role in spinal mobility, a reduction in stress on the intervertebral discs adjacent to the distal anchors may be more beneficial for maintaining spinal mobility, thereby enhancing long-term clinical outcomes. However, this finding is only a hypothesis based on the FEA results and requires further clinical research for validation.

### 4.4. Clinical Implications

The biomechanical findings of this study provide a rationale for the clinical application of HT in selected sEOS patients. From a clinical perspective, the improved stress distribution with HT may translate into several long-term benefits. First, reduced peak stress at the anchors could lower the risk of screw loosening and rod fracture, potentially decreasing the need for unplanned reoperations. Second, although apical fusion arrests growth at the osteotomy site, the overall spinal height at maturity in HT-treated patients was comparable to TDGR in our clinical cohort, suggesting that the initial height gain compensates for the loss of apical growth. Third, the reduced intradiscal stress at junctional zones may contribute to preserving intervertebral disc health, though this requires longitudinal MRI-based follow-up. Finally, by achieving better deformity correction and reducing complication rates, HT may allow more patients to avoid definitive spinal fusion at skeletal maturity, as suggested by our clinical graduation outcomes [24].

### 4.5. Strengths

This study has several notable strengths. First, to our knowledge, this is the first study to directly compare the biomechanical profiles of HT and TDGR using both FEA and in vitro experiments in highly homogeneous sEOS models. The combination of computational and experimental approaches provides complementary evidence that strengthens the validity of our conclusions. Second, the use of identical distraction distances and controlled experimental conditions minimised confounding variables, allowing for a direct comparison of the two techniques. Third, the FEA simulation incorporated spinal growth modelling based on established mechanobiological principles [13,20,21], which more closely replicates the clinical scenario of growing rod treatment than static models. Fourth, the excellent agreement between FEA predictions and in vitro experimental results adds confidence to our findings.

### 4.6. Limitations and Future Directions

For the first time, the present study utilised FEA and in vitro biomechanical experiments to compare the differences in clinical outcomes and biomechanics between TDGR and HT in highly homogeneous scoliotic models, which provided solid evidence for the potential biomechanical advantages of HT over TDGR. However, the study has the following limitations. First, human tissue is complex. Although follower loading was used to simulate the effects of the paraspinal muscles, it still cannot fully simulate the actual role of paraspinal muscles on the spine. Additionally, a formal mesh convergence study was not performed, though the mesh density was comparable to or higher than prior validated studies. A sensitivity analysis of the growth parameter β was not conducted; therefore, the quantitative growth predictions should be interpreted with caution. However, because the same model assumptions were applied to both TDGR and HT, the comparative conclusions remain robust. Second, the specimens used in the in vitro biomechanical experiments were animal spinal specimens rather than human spinal specimens. Obtaining fresh-frozen paediatric cadaveric spines is extremely challenging due to ethical and practical limitations, and ovine models are commonly used as a surrogate. While previous studies have reported general similarities in bone mineral density and segmental stiffness between sheep and human spines [34], important differences must be acknowledged. Ovine vertebrae are smaller in size, have different trabecular architecture, and exhibit a greater disc height-to-vertebral body height ratio compared to paediatric human spines. Moreover, growth plate morphology and closure patterns differ between species. Consequently, the absolute stress and strain values derived from the ovine model should be interpreted as directional trends rather than direct quantitative predictions for paediatric patients. However, the primary goal of this study was to compare two surgical techniques (TDGR vs. HT) under identical, controlled conditions. Because the same ovine specimens, instrumentation, and testing protocols were used for both groups, the relative differences observed between TDGR and HT are likely valid and clinically informative, even if the absolute values differ from the human scenario. Future studies should include: (1) prospective clinical studies with larger sample sizes to validate the biomechanical advantages of HT observed in this study; (2) long-term follow-up studies to assess whether the reduced implant stress with HT translates into lower rates of mechanical complications in clinical practice; (3) MRI-based longitudinal evaluation of intervertebral disc health in noninstrumented segments adjacent to HT constructs; and (4) patient-specific FEA modelling to identify which sEOS patients would benefit most from HT versus TDGR based on individual anatomical and biomechanical characteristics.

## 5. Conclusions

In conclusion, this combined FEA and in vitro biomechanical study demonstrated three main findings. First, compared with TDGR, HT achieved superior initial correction and better maintained correction during follow-up while effectively preserving spinal height. Second, by redistributing stress from the proximal and distal anchors to the apical region, HT significantly reduced peak stresses on the instrumentation and junctional intervertebral discs. Third, although apical fusion arrests growth at the osteotomy site, the overall spinal height at the end of follow-up remained greater in the HT group, suggesting that the initial height gain compensates for the loss of apical growth. These biomechanical advantages support HT as a viable alternative to TDGR in selected sEOS patients, though long-term clinical validation is warranted.

## Figures and Tables

**Table 1:** Comparison of radiological parameters between TDGR and HT.

	TDGR	TDGR (Correction Rate, %)	HT	HT Correction Rate (%)
Main curve (°)				
Preoperative	82.4	-	82.4	-
Postoperative	52.5	36.3%	31.0	62.4%
First growth	60.3	-	33.9	-
First distraction	42.4	48.5%	21.8	73.5%
Second growth	51.8	-	26.3	-
Second distraction	38.8	52.9%	17.0	79.4%
AVT (mm)				
Preoperative	29.65	-	29.65	-
Postoperative	19.25	35.1%	10.45	64.8%
First growth	24.90	-	13.89	-
First distraction	15.42	48.0%	6.35	78.6%
Second growth	18.36	-	9.12	-
Second distraction	12.43	58.1%	5.96	79.9%
GK (°)				
Preoperative	42.9	-	42.9	-
Postoperative	30.0	30.1%	25.2	41.3%
First growth	34.8	-	26.7	-
First distraction	26.6	38.0%	25.6	40.3%
Second growth	32.1	-	28.5	-
Second distraction	29.3	31.7%	25.1	41.5%
T1–L5 height (mmm)				
Preoperative	340.36	-	340.36	-
Postoperative	352.94	-	366.79	-
First growth	359.07	-	371.74	-
First distraction	365.87	-	377.06	-
Second growth	372.55	-	382.76	-
Second distraction	377.87	-	387.04	-

TDGR, traditional dual-growing rods; HT, hybrid technique; AVT, apical vertebral translation; GK, global kyphosis.

**Table 2:** Comparison of correction outcomes between TDGR and HT.

	TDGR	Correction Rate (%) (TDGR)	HT	Correction Rate (%) (HT)	*p* Value
Main curve (°)					
Preoperative	80.92 ± 4.10	-	81.75 ± 3.53	-	0.738
Postoperative	38.97 ± 3.23	51.8%	24.58 ± 2.80	69.9%	<0.001 *
AVT (mm)					
Preoperative	63.73 ± 12.79	-	65.13 ± 13.96	-	0.872
Postoperative	13.15 ± 3.58	79.4%	8.87 ± 1.64	86.4%	0.035 *
T1-L5 height (cm)					
Preoperative	50.15 ± 1.60	-	49.78 ± 1.96	-	0.752
Postoperative	52.00 ± 2.08	-	53.62 ± 1.66	-	0.204
Change	1.85 ± 0.72	-	3.83 ± 0.45	-	<0.001 *

TDGR, traditional dual-growing rods; HT, hybrid technique; AVT, apical vertebral translation. * Indicated significance.

**Table 3:** Comparison of strain in rods between TDGR and HT.

	TDGR	HT	*p* Value
Lower left rod			
Initial surgery	908.83 ± 20.44	929.67 ± 18.02	0.118
Loading (100 N)	1003.33 ± 18.70	991.83 ± 20.94	0.381
Change	94.50 ± 13.26	62.17 ± 17.82	0.010 *
Lower right rod			
Initial surgery	887.17 ± 17.53	913.33 ± 22.99	0.071
Loading (100 N)	1026.33 ± 24.86	996.83 ± 15.00	0.046 *
Change	139.17 ± 23.53	83.50 ± 21.37	0.003 *
Upper left rod			
Initial surgery	1218.50 ± 42.50	1271.00 ± 49.74	0.103
Loading (100 N)	1578.00 ± 50.85	1501.17 ± 47.78	0.034 *
Change	359.50 ± 20.27	230.17 ± 17.26	<0.001 *
Upper right rod			
Initial surgery	1237.67 ± 43.30	1287.00 ± 45.79	0.111
Loading (100 N)	1556.67 ± 41.23	1494.33 ± 42.46	0.040 *
Change	319.00 ± 12.22	207.33 ± 15.22	<0.001 *

TDGR, traditional dual-growing rods; HT, hybrid technique. * indicated significance.

## Data Availability

The original contributions presented in this study are included in the article/Supplementary Materials. Further inquiries can be directed to the corresponding authors.

## Supplementary Materials

The following supporting information can be downloaded at https://www.mdpi.com/article/10.3390/jcm15145352/s1, Figure S1: Standing whole-spine anteroposterior (AP) and lateral radiographs. The Cobb angle of the main curve was 84.3° and the general kyphosis was 42.9°; Figure S2: Model validation. A-B, The comparison of the geometric morphology between the finite element model (82.4°) and the full-spine anteroposterior X-ray (84.3°), with a difference of only 1.9°. C, The comparison of centroid distance between the finite element model and the lateral bending radiograph. D, The flexion-extension, lateral bending, and axial rotation ranges of motion for the T1-T4 segments in the present study were 6.4°, 6.9°, and 7.4°, respectively. These values are comparable to the corresponding values reported by Busscher et al., which were 6.0°, 6.1°, and 7.2° [15]. E, The flexion-extension, lateral bending, and axial rotation ranges of motion for the L1-L4 segments in the present study were 5.7°, 6.2°, and 2.8°, respectively. These values are comparable to those reported by Busscher et al., which were 5.1°, 5.9°, and 2.2° [15]. F, Under conditions of vertical loading, flexion, extension, lateral bending, and rotation, the average stiffness values for the T12-L2 segment in this study were calculated as 9.52 kN/cm, 2.51 N·m/°, 2.68 N·m/°, 4.41 N·m/°, and 1.91 N·m/°, respectively. In previous studies, the reported ranges of average stiffness for T12-L2 were 4.95–12.20 kN/cm, 1.10–4.28 N·m/°, 2.21–4.97 N·m/°, 1.88–2.88 N·m/°, and 1.10-6.01 N·m/° [16,17,35,36]; Figure S3: Schematic diagram of in vitro biomechanical testing; Figure S4: A, In vitro modeling of severe scoliosis; B, TDGR simulation; C, HT simulation.; Figure S5: A-E, For TDGR, the maximum biomechanical stress at the anchors after initial correction, 1st growth, 1st distraction, 2nd growth, and 2nd distraction were 516.69 MPa, 487.36 MPa, 673.10 MPa, 538.91 MPa, and 739.91 MPa, respectively. F-J, For HT, the maximum biomechanical stress at the anchors after initial correction, 1st growth, 1st distraction, 2nd growth, and 2nd distraction were 421.14 MPa, 376.74 MPa, 515.62 MPa, 420.18 MPa, and 662.39 MPa, respectively.; Figure S6: A-E, For TDGR, the maximum biomechanical stress at the rods after initial correction, 1st growth, 1st distraction, 2nd growth, and 2nd distraction were 230.79 MPa, 192.50 MPa, 274.69 MPa, 246.01 MPa, and 321.45 MPa, respectively. F-J, For HT, the maximum biomechanical stress at the rods after initial correction, 1st growth, 1st distraction, 2nd growth, and 2nd distraction were 142.27 MPa, 128.66 MPa, 199.95 MPa, 173.77 MPa, and 227.45 MPa, respectively.; Table S1: Material properties [14]; Table S2: The loads on each vertebra [23]; Table S3: Effect sizes (Cohen’s d) and 95% confidence intervals for in vitro biomechanical comparisons (*n* = 6 per group); Table S4: Comparison of the maximum biomechanical stresses at the anchors and apical vertebrae between TDGR and HT (MPa); Table S5: Comparison of the maximum biomechanical stresses at the intervertebral discs in the junctional zone between TDGR and HT (MPa); Table S6: Comparison of the maximum biomechanical stresses at the anchors between TDGR and HT (MPa); Table S7: Comparison of the maximum biomechanical stresses at the rods between TDGR and HT (MPa).
