# Supplementary material for: Biomechanical Comparison of Hybrid Technique and Traditional Dual-Growing Rods Alone in the Treatment of Severe Early Onset Scoliosis
Source: J Clin Med. 2026 Jul 8;15(14):5352. doi: 10.3390/jcm15145352 (PMC13411218; doi:10.3390/jcm15145352)
Supplement: Supplementary file 1 [file jcm-15-05352-s001.zip › jcm-4338628-supplementary.pdf]

Supplementary materials

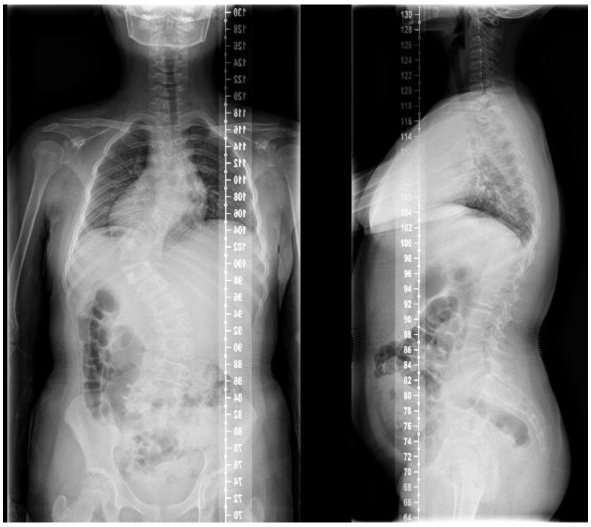

Figure S1 Standing whole-spine anteroposterior (AP) and lateral radiographs. The Cobb angle of the main curve was 84.3° and the general kyphosis was 42.9°.

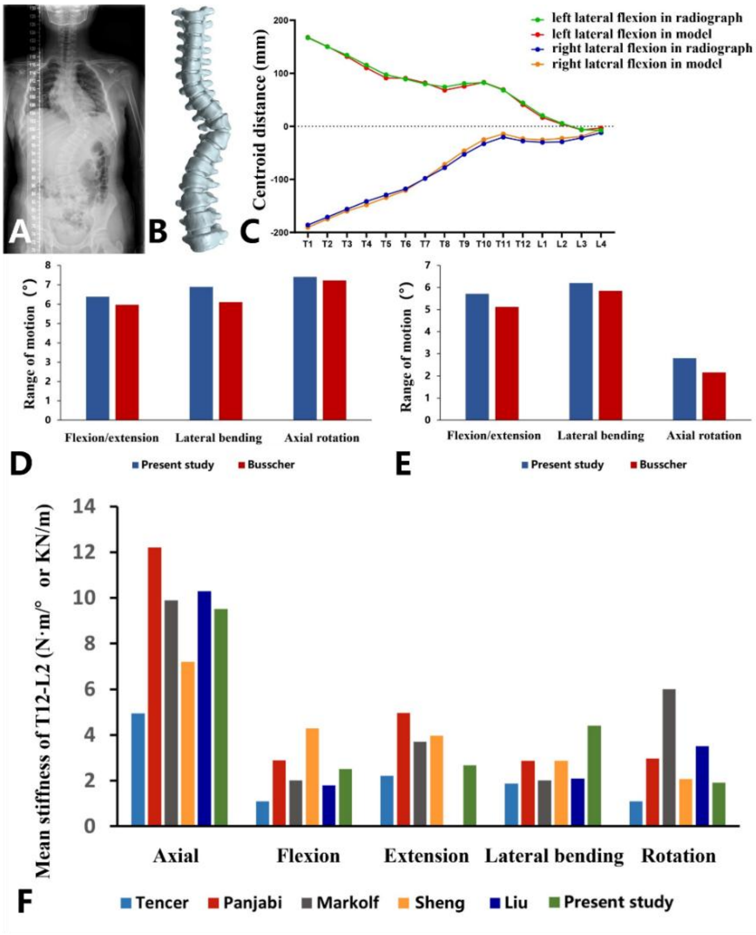

Figure S2 Model validation. **A-B**, The comparison of the geometric morphology between the finite element model (82.4°) and the full-spine anteroposterior X-ray (84.3°), with a difference of

only 1.9°. **C**, The comparison of centroid distance between the finite element model and the lateral bending radiograph. **D**, The flexion-extension, lateral bending, and axial rotation ranges of motion for the T1-T4 segments in the present study were 6.4°, 6.9°, and 7.4°, respectively. These values are comparable to the corresponding values reported by Busscher et al., which were 6.0°, 6.1°, and 7.2°[15] . **E**, The flexion-extension, lateral bending, and axial rotation ranges of motion for the L1-L4 segments in the present study were 5.7°, 6.2°, and 2.8°, respectively. These values are comparable to those reported by Busscher et al., which were 5.1°, 5.9°, and 2.2°[15] .**F**, Under conditions of vertical loading, flexion, extension, lateral bending, and rotation, the average stiffness values for the T12-L2 segment in this study were calculated as 9.52 kN/cm, 2.51 N·m/°, 2.68 N·m/°, 4.41 N·m/°, and 1.91 N·m/°, respectively. In previous studies, the reported ranges of average stiffness for T12-L2 were 4.95-12.20 kN/cm, 1.10-4.28 N·m/°, 2.21-4.97 N·m/°, 1.88-2.88 N·m/°, and 1.10-6.01 N·m/°[16–17,35,36].

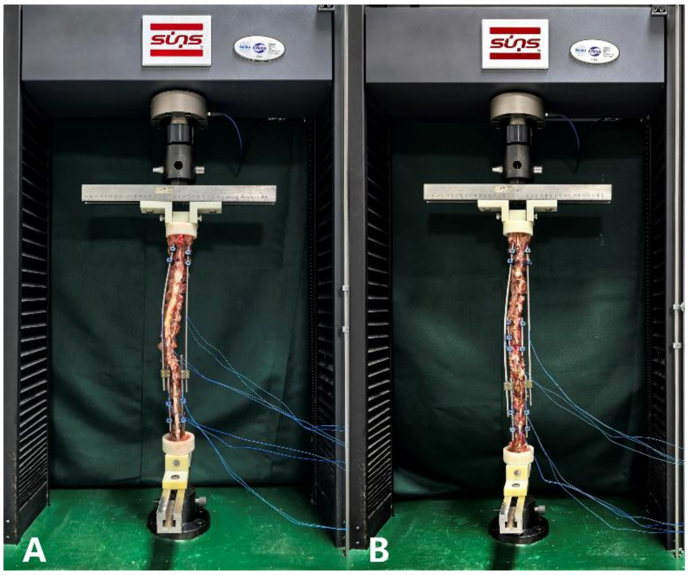

Figure S3 Schematic diagram of in vitro biomechanical testing

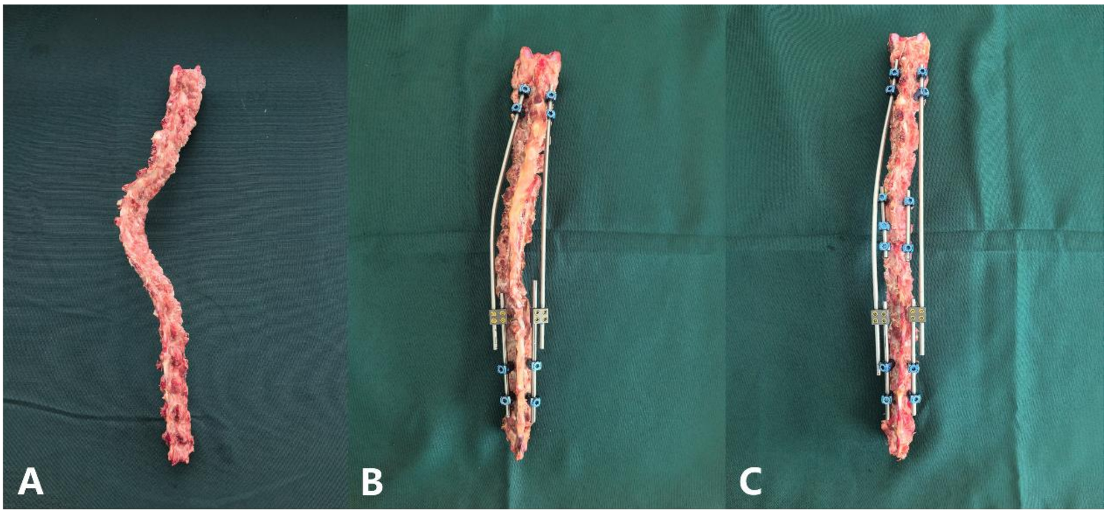

Figure S4 A, In vitro modeling of severe scoliosis; **B**, TDGR simulation; **C**, HT simulation.

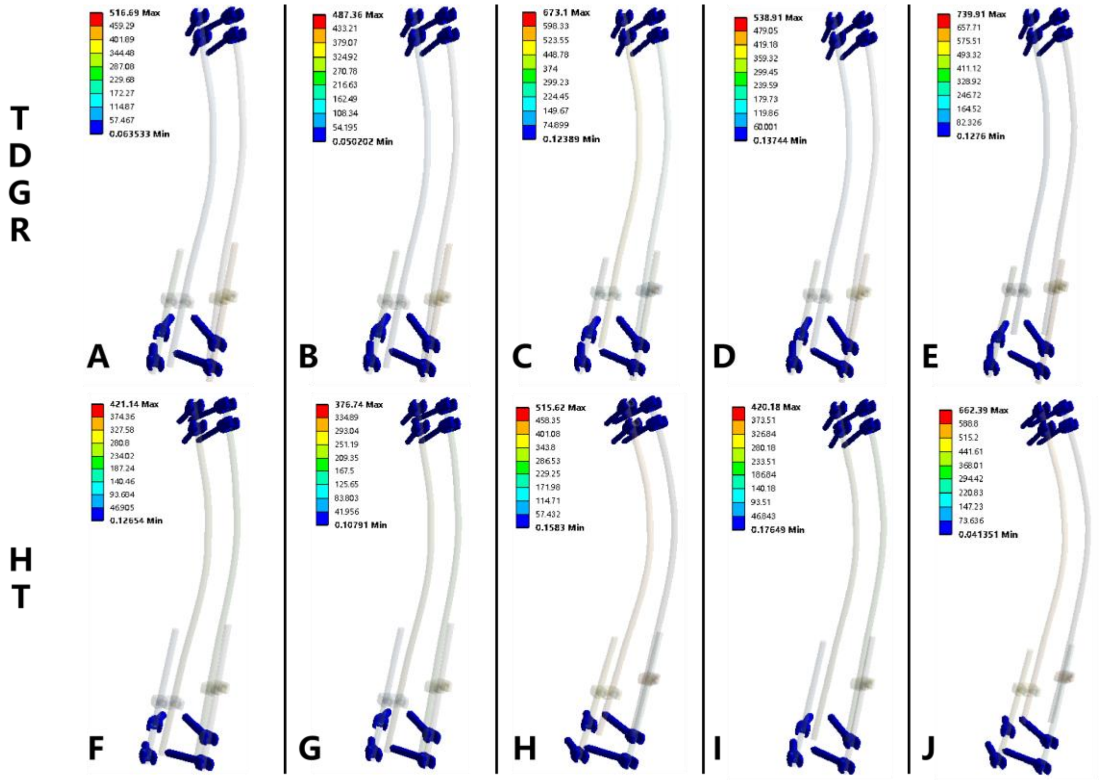

Figure S5 A-E, For TDGR, the maximum biomechanical stress at the anchors after initial correction, 1st growth, 1st distraction, 2nd growth, and 2nd distraction were 516.69 MPa, 487.36 MPa, 673.10 MPa, 538.91 MPa, and 739.91 MPa, respectively. **F-J**, For HT, the maximum biomechanical stress at the anchors after initial correction, 1st growth, 1st distraction, 2nd growth, and 2nd distraction were 421.14 MPa, 376.74 MPa, 515.62 MPa, 420.18 MPa, and 662.39 MPa, respectively.

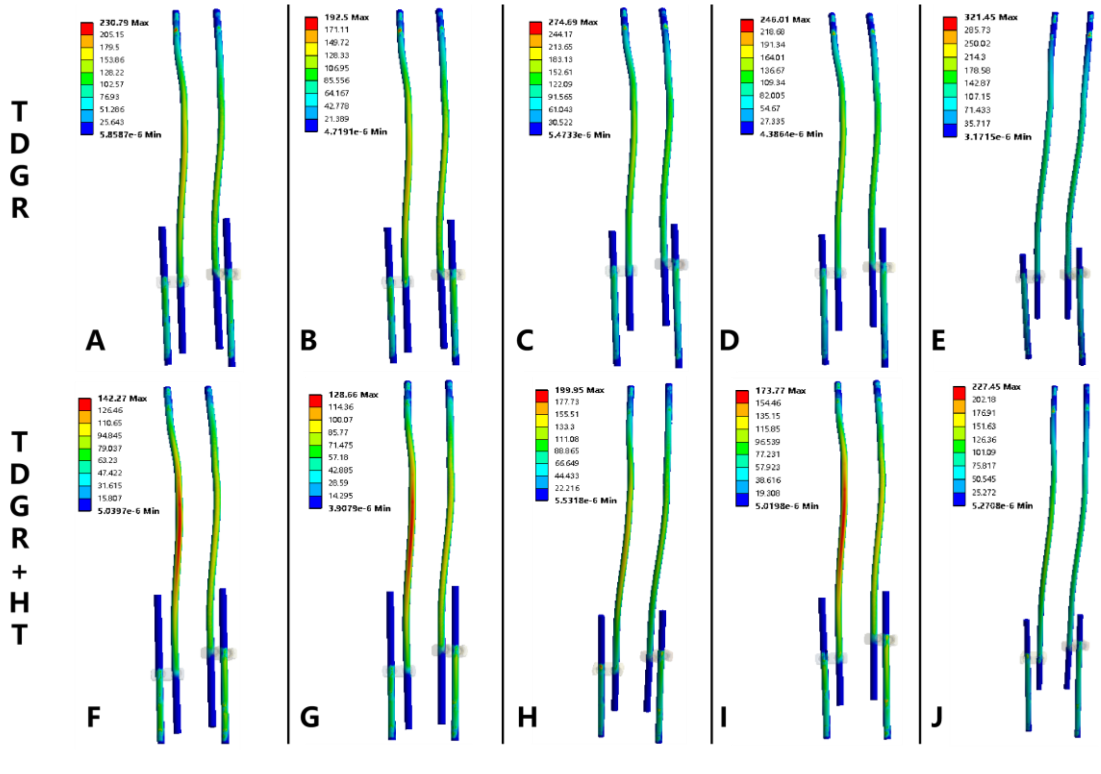

Figure S6 A-E, For TDGR, the maximum biomechanical stress at the rods after initial correction, 1st growth, 1st distraction, 2nd growth, and 2nd distraction were 230.79 MPa, 192.50 MPa, 274.69 MPa, 246.01 MPa, and 321.45 MPa, respectively. **F-J**, For HT, the maximum biomechanical stress at the rods after initial correction, 1st growth, 1st distraction, 2nd growth, and 2nd distraction were 142.27 MPa, 128.66 MPa, 199.95 MPa, 173.77 MPa, and 227.45 MPa, respectively.

Table S1 Material properties[14].

Element	Elasticity modulus (MPa)	Poisson ratio	Cross-sectional area (mm^2)	Element Type
Cortical bone	12000	0.300	-	Quadrilateral
Cancellous bone	500	0.200	-	Quadrilateral
Annulus fibrosus	4.2	0.300	-	Quadrilateral
Nucleus pulposus	2	0.499	-	Quadrilateral
Growing rod	113800	0.342	-	Hexahedron
ALL	20	0.300	38.0	
PLL	70	0.300	20.0	
Joint capsule	20	0.300	40.0	
LF	50	0.300	60.0	Spring
ISL	28	0.300	35.5	
SSL	28	0.300	35.5	
ITL	50	0.300	10.0	

ALL, anterior longitudinal ligament; PLL, posterior longitudinal ligament; LF, ligamentum flavum; ISL, interspinous ligament; SSL, supraspinous ligament; ITL, intertransverse ligament.

Table S2 The loads on each vertebra[23].

Segment	Percentage of body weight (%)	Loading (N)
T1	1.1+8.0 (head)	40.84
T2	1.1	4.94
T3	1.3+4.0 (upper limb)	23.79
T4	1.3+4.0 (upper limb)	23.79
T5	1.3+4.0 (upper limb)	23.79
T6	1.3	5.83
T7	1.4	6.28
T8	1.5	6.73
T9	1.6	7.18
T10	2.0	8.98
T11	2.1	9.43
T12	2.5	11.22
L1	2.4	10.77
L2	2.4	10.77

L3	2.3	10.32
L4	2.6	11.67
L5	2.6	11.67
Total	50.8	228.01

Table S3 Effect sizes (Cohen's d) and 95% confidence intervals for in vitro biomechanical comparisons (n = 6 per group).

Variable	Mean Difference (TDGR – HT)	Pooled SD	Cohen's d	95% CI	Effect size interpretation
Postoperative main curve (°)	14.39	3.02	4.76	[2.54, 6.98]	Very large
Postoperative AVT (mm)	4.28	2.78	1.54	[0.18, 2.90]	Large
Increase in T1-L5 height (cm)	-1.98	0.60	-3.30	[-5.21, -1.39]	Very large
Lower left rod – strain change (με)	32.33	15.71	2.06	[0.60, 3.52]	Large
Lower right rod – strain change (με)	55.67	22.48	2.48	[0.94, 4.02]	Large
Upper left rod – strain change (με)	129.33	18.82	6.87	[3.95, 9.79]	Very large
Upper right rod – strain change (με)	111.67	13.81	8.09	[4.75, 11.43]	Very large

Note: Cohen's d interpretation: small (0.2–0.5), medium (0.5–0.8), large (>0.8). Negative d indicates HT superior to TDGR (greater height gain). All comparisons were statistically significant ($P < 0.05$).

Table S4 Comparison of the maximum biomechanical stress at the anchors and apical vertebrae between TDGR and HT (MPa).

		Initial surgery	1st growth	1st distraction	2nd growth	2nd distraction
T3	TDGR	29.29	25.92	34.20	29.03	41.54
	HT	23.15	19.13	26.19	22.36	36.38
T4	TDGR	10.35	10.10	15.35	11.37	19.56
	HT	7.12	6.17	9.88	7.14	11.57
T9	TDGR	1.48	1.42	2.80	1.82	3.17
	HT	13.86	11.15	17.91	11.77	25.63
T10	TDGR	2.48	2.39	3.36	2.49	4.03
	HT	2.28	1.46	2.64	1.52	2.86
T11	TDGR	3.04	2.81	4.24	2.71	4.75
	HT	15.24	13.18	16.15	12.96	16.57

L3	TDGR	15.52	14.66	25.96	16.09	29.80
	HT	12.11	11.29	12.87	11.40	13.24
L4	TDGR	15.48	13.17	29.33	17.27	34.29
	HT	12.25	11.48	15.59	12.46	18.52

Table S5 Comparison of the maximum biomechanical stress at the intervertebral discs in the junctional zone between TDGR and HT (MPa).

		Initial surgery	1st growth	1st distraction	2nd growth	2nd distraction
T2/3	TDGR	2.00	1.93	2.85	2.20	3.22
	HT	1.11	1.09	1.22	1.37	1.62
L4/5	TDGR	3.03	2.46	3.95	3.60	4.25
	HT	1.53	1.30	1.77	1.70	2.04

Table S6 Comparison of the maximum biomechanical stress at the anchors between TDGR and HT (MPa).

		Initial surgery	1st growth	1st distraction	2nd growth	2nd distraction
T3 left	TDGR	315.60	303.02	427.49	363.72	567.56
	HT	243.34	239.51	380.61	290.60	403.70
T4 left	TDGR	253.79	248.94	388.66	304.08	428.36
	HT	237.10	199.27	280.98	234.49	323.68
T3 right	TDGR	515.17	468.86	551.99	486.30	602.01
	HT	390.29	376.74	466.29	413.32	500.56
T4 right	TDGR	437.77	360.61	473.50	421.14	528.00
	HT	304.63	263.55	385.92	325.39	427.78
L3 left	TDGR	502.04	434.38	571.86	499.70	639.80
	HT	377.86	303.55	436.34	359.81	485.28
L4 left	TDGR	516.69	487.36	673.10	538.91	739.91
	HT	421.14	347.61	515.62	420.18	662.39
L3 right	TDGR	445.55	320.89	484.34	370.92	579.16
	HT	275.15	228.07	324.58	261.42	384.06
L4 right	TDGR	499.52	434.29	495.86	458.93	593.95
	HT	366.10	341.95	437.65	403.56	626.58

Table S7 Comparison of the maximum biomechanical stress at the rods between TDGR and HT (MPa).

Rod		Initial surgery	1st growth	1st distraction	2nd growth	2nd distraction
Lower left	TDGR	144.90	129.30	152.44	141.56	153.40
	HT	101.25	81.57	168.21	140.28	179.44
Lower right	TDGR	164.34	143.01	173.79	162.03	256.91
	HT	114.05	99.67	145.95	110.51	165.91
Upper left	TDGR	230.79	192.50	274.69	246.01	321.45
	HT	142.27	128.66	199.95	173.77	227.45

Upper right	TDGR	168.71	149.75	219.69	193.20	235.73
	HT	116.78	102.67	127.07	104.05	173.28
